# Exploring strategies to promote influenza vaccination of children with medical comorbidities: the perceptions and practices of hospital healthcare workers

**DOI:** 10.1186/s12913-019-4742-5

**Published:** 2019-11-29

**Authors:** Vanessa Ma, Pamela Palasanthiran, Holly Seale

**Affiliations:** 1 Undergraduate Medicine Program, Faculty of Medicine, University of New South Wales, Sydney, NSW Australia; 2 Sydney Children’s Hospitals Network, Randwick, NSW Australia; 3 School of Women’s and Children’s Health, University of New South Wales, Sydney, Australia; 4 School of Public Health and Community Medicine, University of New South Wales, Sydney, NSW Australia

**Keywords:** Influenza, Vaccination, Paediatric, Comorbidities, Healthcare worker, Promotion

## Abstract

**Background:**

To explore how the influenza vaccine is promoted and delivered to children with medical comorbidities in the hospital setting, as well as the facilitators of and barriers to vaccination from the healthcare worker perspective.

**Methods:**

Semi-structured interviews were conducted with staff members (*n* = 17) at a paediatric hospital in Sydney, Australia between April and July 2018. This included nurses, clinical nurse consultants, pediatricians and department heads. The interviews were transcribed and analysed iteratively to generate the major themes.

**Results:**

Approaches used to promote and/or deliver the influenza vaccine varied among the participants. Some described the vaccine as an ingrained component of their clinical consultation. Others acknowledged that there was missed opportunities to discuss or provide the vaccine, citing competing priorities as well as a lack of awareness, time and resources. Participants perceived that some parents had concerns about safety and appropriateness of the vaccine for their child. While there was some support for sending reminders and/or educating patients through the hospital, there were differing perspectives on whether tertiary centres should be delivering the vaccine.

**Conclusion:**

Hospital-based interventions to increase vaccine uptake must consider the needs of staff. Easily accessible information and increased awareness of the recommendations among staff may lead to improved uptake in this hospital. Additional resources would be required to increase on-site delivery of the vaccine.

## Background

In Australia, high-risk children (HRC) including those with medical comorbidities, aged 6 months and greater are recommended to receive the influenza vaccine each year [1]. The vaccine is funded under the National Immunisation Program and has been freely available to this group since 2010 [2]. Nevertheless, vaccine coverage is suboptimal. Estimates among hospitalised HRC range from 5 to 26.9% [3–5], with a higher Fig. (41%) reported in a survey conducted in two Sydney hospitals [6]. A recent survey in 2017 across three large paediatric hospitals (located in Western Australia, Victoria and Queensland) documented the uptake at 52% for children with medical comorbidities, however this study was conducted during an unusually high burden influenza season [7].

Health care workers (HCWs) play a pivotal role in vaccine uptake. It is well documented that an HCW recommendation is a strong predictor of influenza vaccination in HRC [6, 8–11]. Physicians are a trusted source of information whom parents rely upon to initiate vaccine conversations [12, 13]. However, recently it was reported that a recommendation from a hospital-based physician is a stronger predictor of influenza vaccination for Australian HRC, compared to a recommendation from a primary care physician [7]. The study also identified that hospital-based physicians were the caregivers’ most commonly reported source of trusted vaccination information. Parents may perceive that their child’s specialist has more detailed knowledge regarding the child’s condition and their treatment regiments. The problem is however that hospital HCWs may lack knowledge regarding the current guidelines/recommendations regarding influenza vaccination [14]. In a survey of pediatricians in two Sydney hospitals, only half (56%) had a correct understanding of the vaccine recommendation for HRC. Furthermore, approximately 14% believed there were restrictions for children under 5 [15], which is significant considering the large morbidity in this age-group [16]. HCWs have also reported difficulties identifying HRC during consultations [17], as individual physicians may be less familiar with the medical history of a high-risk child, especially if he/she sees multiple providers [18].

HRC have frequent contact with hospitals and secondary care teams [19, 20]. Nevertheless, missed opportunities to vaccinate occur in hospitals; survey data from two Sydney hospitals, for instance, indicated that unvaccinated HRC attended a median of three outpatient visits during the influenza season in which they did not receive the vaccine [6]. Children often obtain the same immunisation status over successive seasons, with those experiencing a missed opportunity to vaccinate in one season being most likely to experience another in the next. Thus, it has been suggested that missed opportunities to discuss or provide the vaccine are not isolated events, but representative of systematic failings in the practices of HCWs [21]. Before we can design new interventions to improve uptake in this population, it is important to understand the current landscape around the promotion and delivery. We conducted a qualitative study involving HCWs at one large Sydney paediatric hospital, to understand what is happening around influenza vaccination of HRC.

## Methods

A qualitative study was conducted involving in-depth interviews with a sample of hospital staff at one Sydney tertiary paediatric hospital. A purposeful, non-probability sampling method was used to recruit HCWs that were: involved in the clinical care of HRC and/or involved in the promotion or delivery of the influenza vaccine to HRC. Recruitment occurred from April – July 2018. Ethics approval was obtained from the Sydney Children’s Hospital Network (LNR/18/SCHN/24) and written informed consent was received from all participants.

In 2018, free influenza vaccination was available in seven of eight Australian jurisdictions to all children ≤5 years of age. As outlined in the introduction, free influenza vaccines had been available for children with certain HRCs as part of the National Immunisation program since 2010. In Australia, the majority of childhood immunisation is provided in the primary care setting, and a smaller proportion delivered via school-based programs. In some of the larger Australian paediatric hospitals, Immunisation Specialist Services or clinics (with dedicated staff) have been operating to provide better access to children with chronic medical conditions. During the influenza season, many of these clinics provide a drop-in service to allow patients and their families member (at a small cost, if they are not eligible) to receive the influenza vaccine. While the study site is a paediatric public (teaching) hospital with an emergency department, it does not currently have a dedicated immunisation clinic. In this hospital, influenza vaccination is provided opportunistically by staff in the outpatient setting.

The researchers liaised with a staff member who worked with HRC at the hospital to identify key stakeholders who met the inclusion criteria. This included department heads, pediatricians, clinical nurse consultants, and nurses. Department heads were contacted firstly and then were asked to pass on the invitation to any staff members they deemed appropriate for the study. Targeted recruitment was also used to ensure that we captured members of the nursing staff that were known to be involved with the current vaccine delivery processes onto the study. HCWs who consented to the study were contacted via email to organise a face-to-face or telephone interview at a convenient time. Participants were also asked to recommend other HCWs who would be suitable for the study (i.e. snowball recruitment). Due to the recruitment process, we were unable to capture any information on staff who declined to participate.

Semi-structured interviews (15–60-min duration) were conducted by researchers who were independent of the hospital. An interview guide (Additional file 1) containing topics of interest was used to guide discussions. Questions asked pertained to the participant’s current practices, the perceived attitudes of parents, challenges in vaccine delivery, and potential interventions that may increase vaccine uptake. Open-ended questions, paraphrasing, and additional questions were used in a flexible interview approach to allow the exploration of a wide range of ideas. Interviews were audio-recorded with the participant’s permission. At the end of each interview, the main ideas expressed were summarised back to the participant to check the researcher’s understanding. No field notes were collected, repeat interviews were not undertaken, nor were transcripts returned to participants. Most interviews were conducted face to face in the hospital by Holly Seale, Senior Lecturer (PhD, MPH, BSc) who has previously undertaken and published qualitative research focused on influenza vaccination. Vanesa Ma, a medical student, received training to undertake in-depth interviews who sat in on the interviews and undertook three of them. There were no established relationships between the research team and the participants prior to the study. At the end of each interview, a preliminary analysis of the data was undertaken by making note of concepts, ideas and potential themes. This process assisted with identifying further areas for exploration in subsequent interviews and assisted with identifying further departments/staff members to contact and recruit. The analysis continued throughout the data collection period, as the team constantly looked at and discussed the ideas arising from the data. All interviews were audio recorded and professionally transcribed verbatim. The researchers used NVivo 12 to code the data [22]. In analysing the data we followed the steps proposed by Braun and Clarke [23], i.e. transcription of interviews, familiarization by reading and re-reading and looking for meanings and patterns for coding in the data, generating initial codes, generating and reviewing themes, and finalizing the analysis. A pre-determined theoretical/conceptual coding framework was not utilised during the analysis process. Rather, the codes arose based on the participants language, perceptions and sentiments. Feedback on the themes was not sought from participants, as we tried to reduce the research burden on the hospital staff involved.

## Results

Seventeen HCWs (9 doctors, 8 nurses) were interviewed between April and July 2018. Participants had worked in healthcare for an average of 28 years (range: 10–56 years) and the majority (14/17) had received the influenza vaccine in 2017. The study results are presented below according to the themes identified:

### Motivation to promote vaccination driven by clinical interactions with influenza positive children

Participants who were motivated to promote the vaccine often spoke of witnessing the impacts of influenza among their paediatric patients. This sentiment repeatedly came from those working in Infectious Disease and Respiratory Medicine Departments. In those settings (where staff members routinely encounter patients with severe respiratory infections), it was suggested that influenza vaccination works “because everyone’s quite motivated to push it” (Participant 8). There was a readiness to speak about influenza unprompted, as well as resolution to continue to discuss the vaccine with parents: “[F] or some people, at least at that first conversation, considering influenza immunisation is a bridge too far … so I’m prepared to bring it up again and see if people are willing to talk about it at a later date.” (Participant 1).

### Hospital HCWs are a trusted source of information

Amongst the participants, it was suggested that it was important for an influenza vaccination recommendation to came from the hospital HCW as it was perceived that parents have high levels of trust in these specialists: “[Patients] get very attached to their specialist … especially people with chronic health issues. They don’t want to go to their GP for a lot of things, stuff that they should even go to their GP [for] … They’re scared to do it [without the specialist’s recommendation].” (Participant 15).

Due to this level of trust and the close relationship formed between providers and families, some participants perceived parents to be very accepting of recommendations including vaccination. As one participant stated, “Once they’re in our service … they trust us. And if we say we think this is a good thing then most of them will do it” (Participant 8).

### Parents are perceived to be accepting of vaccination but can have reservations about safety

While acknowledging that the parents existed on a “spectrum” (Participant 16) when it came to vaccine acceptance, it was perceived that amongst parents of HRC, encouraging parents to get their HRC vaccinated “[didn’t] take a lot of persuasion” (Participant 15). It was noted that ‘anti-vaxxers’ are “a very small proportion” (Participant 2) of patients, with few participants having experiences with any “violent negativity” (Participant 1).

“I can’t think of a single person who’s not wanted to have the vaccine … after [the] explanation.” (Participant 17)

“They’ve already been hit by a chronic condition which is life-limiting and it’s like ‘What can we do? … Antibiotics: we’ll do it. Immunisation: we’ll do it.’” (Participant 6).

It was suggested that there was greater awareness and acceptance amongst parents whose children have more serious health conditions. Whereas for other parents, they can be unaware about the child’s eligibility to receive certain immunisations: “[S] ome patients … get it done every year; they’re knowledgeable about why they need it. I think other patients are surprised when you recommend it.” (Participant 4).

Concerns about safety and effectiveness were suggested as being common reasons for hesitancy, as were misconceptions surrounding the rational and need for influenza vaccination. Additionally, reluctance was reported for children who are prone to seizures due to fears they would “get a temperature with the vaccine” (Participant 4).

“[W] e have that constant battle with the families and their perception of the safety or the sequelae of being vaccinated. So, I spend a big chunk of clinic just convincing people.” (Participant 6)

### Staff members have difficulties with keeping up to date with recommendations

Participants expressed difficulties in keeping up with the recommendations stating, “[They] keep changing [and] … as clinicians … it’s not at the top of your head until it’s there” (Participant 3). Others were concerned about the distribution of knowledge, particularly among HCWs with limited exposure to influenza or infectious diseases: “I think people who work in the field tend … to have more knowledge about who is at risk, … what vaccines are available, and how to access them. But that’s not everyone who needs to be reached. There are many more providers than that who are responsible for recommending and providing [the] … vaccine.” (Participant 1).

These issues were exacerbated by a perceived lack of direction. Participants felt there was little guidance on vaccine issues and were unsure where to acquire information: “There’s no one person to coordinate … and there’s no one person that deals with immunisations … People are sort of going around [asking] ‘What’s happening with the flu vaccine, and what is it?... Is it quadrivalent? … Is it activated?’” (Participant 15).

They highlighted the fact that staff immunisations are “really pumped up” (Participant 3) in comparison to patient immunisations. Indeed, they did not recall receiving material from the hospital. During these discussions, no references were made to any formal procedural guidelines within the hospital. Instead, participants highlighted a lack of governance over the issue and the subsequent need for self-direction. Indeed, there was agreement that the departments were largely siloed in their actions.

### Conflicting priorities in terms of time and resources

For some participants, the vaccine was considered an ingrained component of their clinical practice: “[I] t doesn’t matter if you see us in January or December … we talk about it as something that [the] child should have, that parents should have on their radar.” (Participant 3). Conversely, there were others who acknowledged forgetting to discuss the vaccine. In contrast to the first group, these participants appeared to place less emphasis on the immunisation. One, for instance, commented “People are either going to do it or not” (Participant 2). The issue of competing priorities was not uncommon when it came to staff members remembering to speak to parents about the vaccine: “We’re dealing with other things … and acute symptoms tend to distract you from issues of more… prophylactic management.” (Participant 9). Conflicting priorities also was linked to the issue of resources that are required for promoting and delivering the vaccine onsite. It was suggested that the influenza vaccine was considered a lesser priority as resources were required for other issues: “I’ve got many other priorities. And I think most of the departments would feel that.” (Participant 2).

### It’s the role of the primary care doctor to deliver immunisation

Perspectives were varied on the role of the hospital in delivering the influenza vaccine. To one participant, the hospital’s responsibility was confined to those who frequently engaged with the hospital system and therefore did not have access to their general practitioner: “I would’ve thought … this is a primary health problem … rather than just opportunistically vaccinating anyone who walks in the door.” (Participant 17).

Another did not consider on-site vaccination to be a significant driver of immunisation among their patients noting, “We have been saying you can go [on-site] to get a flu shot, but most of them say ‘Oh I’ll just go get it at my GP’ because it’s free for … the child” (Participant 11). Amongst those who recommended their patients receive the vaccine in the primary care setting, they did acknowledge that there was an issue with keeping track of vaccine uptake as records were not kept for off-site vaccination, nor were children followed up. However, there were other participants who suggested that on-site vaccination was considered highly beneficial to uptake among HRC. Hospital patients were described as a “captive audience” (Participant 17) who were “more likely to act” (Participant 5) if they were offered the vaccine. Conversely, referring a patient to their general practitioner may result in a missed opportunity:“If [patients] go away and think about it, they’ll forget about it … [T] he message is lost and the opportunity to actually give it is then lost.” (Participant 5).

While general practitioners were acknowledged as the drivers of immunisation in the community, participants expressed concerns about relying on primary care. There was doubt the vaccine was being recommended to all necessary at-risk groups: “[The] patient groups who kind of flitter in and out of hospital … I don’t think that they get the same information from their GP to … [vaccinate] independently of the hospital.” (Participant 6).

Participants had also received reports from parents that some general practitioners were reluctant to administer the vaccine due to the child’s underlying condition: “[T] he GPs often say, ‘Ask the Doctor if it’s okay’. So, it’s the hesitancy of safety.” (Participant 11). Staff members indicated a need for a greater connection between hospitals and primary care to ensure a uniform message about the influenza vaccine was reaching the public: “[I] f we’re not going to have … this paradoxical, see-saw effect between what the hospitals are saying and what the community is hearing, we have to get the GPs on board.” (Participant 6).

### The outpatient department could be used more effectively to vaccinate HRC

Participants who vaccinated on-site tended to provide the immunisation through the Outpatients Department. In-patient vaccinations were generally uncommon as “the priority is [the patient’s] clinical care right there and then” (Participant 3). Several also believed it would be difficult to vaccinate on the ward due to the child’s health status: “I don’t think [vaccination is] practical when you’ve got an acute illness.” (Participant 7).

While there was some level of satisfaction with the current outpatient system, others believed it lengthened consultations. One participant felt unsupported in the current set-up, suggesting that it placed additional pressure on the doctor. They proposed that immunising patients in the waiting room would allow “flow and efficiency” (Participant 6). It was suggested that “people … who have had their flu vaccine … every time they come to my clinic; they don’t need to be seen by me. You ask the parent directly, … ‘Do you want your child to be immunised against the flu?’ … Get that going while they’re in the waiting room.” (Participant 6).

Several practical barriers to the delivery of the vaccine in outpatients were highlighted including a lack of time, staff and dedicated space: “I’ve been short-staffed and the … staff that I get aren’t accredited for immunisations … I don’t have enough rooms … Space is an issue.” (Participant 14). Another key issue was that in the outpatient setting, children may not necessarily be primed for receiving a vaccine. For some HRC, a visit to an outpatient setting may be for a general consultation which may not necessarily involve blood taking or other invasive procedures. In these situations, it may be important to encourage parents to prepare (prime) their child to the possibility of receiving a vaccine during an outpatient visit.

“[F] or those patients who … [have] been promised by their parents that today … they don’t need any blood tests. And then, for the parent … to have to break that promise … I think they are … the ones that would normally prefer to go to their GP” (Participant 4).

### Increasing awareness amongst providers and parents

Increasing awareness about the recommendations for influenza vaccination and the onsite strategies to vaccinating children was suggested as first steps to improve practices. As one participant stated: “I didn’t know how to [vaccinate] here, so … that’s why I’ve been sending [patients back to their] GP” (Participant 11). Several participants were in favour of sending messages to parents, reminding them to vaccinate their child against influenza. As highlighted by one, “Anything you could do to make it easier for families, easier for staff, to remember to get the vaccination … is a good thing” (Participant 1). Both text messages and emails were considered viable methods of distributing information. Another suggestion involved including reminders with the appointment letter and sending a letter to the patient’s general practitioner. Participants were also willing to send messages through existing channels, such as newsletters and websites, if provided with the information. Others felt visual reminders would be valuable stating, “[I] t has to be a visual cue because there’s just too much going on” (Participant 13).

Nevertheless, there was concern about providing mass information as HRC may have conditions which impact the timing and appropriateness of their vaccinations: “I think you’d still need to be mindful of who you’re sending [reminders] to in case there is some complicating issue. Because … they’re quite rare diseases … so I don’t think you could just do a blanket one-size-fits-all [message].” (Participant 15).

Others felt an alert system would be beneficial, with one noting, “[T] he moment you see a patient, you’re going to look up their medical records and if some sort of alert came up … [saying the] seasonal flu vaccine is available … it’d be useful … because there’s a lot going on” (Participant 15).

In terms of communication, several participants considered their colleagues capable of navigating discussions and did not believe additional training would be necessary. One, however, felt extra training would benefit HCWs with limited exposure to influenza and infectious diseases stating, “Although we’re paediatricians, we’re quite far removed from infectious diseases and we don’t do a lot of management” (Participant 13). Another believed it could increase confidence in less experienced clinicians noting, “The first medical point of contact parents will have will be with the registrar or the resident … [and] they would probably feel less comfortable with a strong-willed parent” (Participant 7).

## Discussion

Given the suboptimal influenza vaccine coverage for children with chronic and underlying medical conditions, this study was undertaken to examine the current landscape around the promotion and delivery of seasonal influenza vaccines at a major paediatric hospital in Sydney. Not surprising that we found that staff members who were responsible for caring for children hospitalised with influenza, were those who were more likely to consider conversations about vaccination as an ‘ingrained’ element of their consultations. They also seemed more willing to have multiple follow-up conversations with parents and had systems in place to get the children vaccinated onsite. This is important as higher coverage has been seen when clinicians continue to nudge parents in spite of resistance [24].

Not all staff members felt that it was the hospital’s responsibility to promote and deliver the influenza vaccination. For some, they preferred to designate responsibility with the primary care physician. Competing priorities during the hospital consultation, lack of resources to deliver vaccination, coupled with the fact that not every HRC will have a hospital consultation during the influenza season were just some of the reasons purported in support of primary care provision. The problem here is threefold: firstly, as previously alluded to, parents are placing higher value on a recommendation from a specialist/hospital physician compared to a primary care physician [7]. Supporting this is the findings from recent Australian studies that found that parents who receive a recommendation from their paediatrician or specialist are 4–16 times more likely to immunise their child [7, 25]. Secondly, general practitioners report being unsure of their role in the management of HRC [26], locating the responsibility of vaccine conversations with the child’s specialist [27]. Lastly, not all children attending the hospital setting may attend primary care regularly or have a regular GP. Merckx et al. [28] for instance, found that almost one-fifth of children immunised in a hospital clinic had no primary care doctor. Even if a family does see a GP, by requiring a child to receive the vaccine in the primary care setting and not immediately onsite, we are adding to the burden of healthcare visits. While these findings support the need for stronger communication between the primary and tertiary care sector and the need to support training of primary care providers to improve their confidence in communicating about vaccination to HRC, they also support the need for hospitals and the staff members to reflect on the key role they could play in supporting improvements in coverage in this at-risk population.

Overwhelmingly, participants spoke about the fact that they did not receive updated information about the influenza vaccine and about recommendations for HRC. This information may have been available but due to conflicting priorities, it had not come onto the staff members radar. Confusion surrounding where to access information within the hospital’s intranet may have contributed to the knowledge gaps in our participants. This is not the first time this finding has been reported. Previously, Philips et al. identified that Australian medical staff are unaware of immunisation resources, including telephone advice lines and government information, highlighting the need for greater promotion of existing tools [29]. Given that HCWs often lack the time and capacity to conduct their own research [30], they require easy access to educational material and resources [31]. The problem is around how to effectively deliver these materials to staff. Posters are often not seen on walls, while printed materials may get sidelined. In this setting, having in-service sessions which including tailored information about the current recommendations, delivered at the department level may assist, in addition to information sessions delivered through-out the influenza season at the hospital level. Having a vaccination champion within each department may also assist with supporting staff to promote and/or deliver vaccine on-site. To support parents to speak up about the vaccine, there is still value in having promotion materials (in a range of languages) at the healthcare facility.

Competing priorities has previously been identified as a barrier to lengthy vaccine conversations by specialists in the United States [31]. This issue was also a common denominator amongst our participant especially amongst HCWs who care for children with highly complex and multiple medical conditions. Competing priorities is not an issue unique to the tertiary sector but also clearly exists for primary care providers [32] as well. In both settings, there is a need for further work to explore how to support providers to value and prioritise discussions about vaccination during their consultations.

One suggestion to assist with the afore mentioned issue is to introduce interventions which targeted parents, such as education and reminder/recall systems. As there is limited time for patient education during consultations, educational tools (e.g. fact sheets) may assist HCWs in communicating the need for vaccination [31]. Martin et al. [33], for instance, employed an asthma education tool during consultations to increase vaccination rates from 37 to 92% among children with persistent asthma. While this study involved only a subset of HRC and is limited by its small sample size, it suggests a strong need to engage parents in vaccine conversations. Conversely, the use of reminder/recall systems in primary care and private practices has achieved only moderate effects [34], with Szilagyi et al. [35] suggesting such methods have a “ceiling” coverage of approximately 30%. Multi-component interventions (i.e. “bundles”) that target both HCWs and parents are thus likely required to achieve optimal coverage within this hospital.

Opportunistic immunisation through the hospital has been shown to increase uptake. Pappano et al. [36], for instance, found higher coverage (57%) among at-risk children who were offered the influenza vaccine in a paediatric emergency department compared to those who were given education on where to obtain the vaccine (36%). Furthermore, while participants in this study were reluctant to vaccinate inpatients, on-ward vaccination programs have been shown to be feasible when there is collaboration with the inpatient clinical team [37]. The provision of influenza immunisation through dedicated vaccination clinics has also been trialled in several Australian hospitals, with a subsequent increase in the number of vaccinations administered [38]. Such interventions, however, often require external funding and the employment of additional staff [28, 39]. A lack of support from other HCWs and minimal incorporation into the local health setting are further barriers to their implementation [40]. Additional resources would be required before increased on-site vaccination is feasible and sustainable in this hospital.

Our study has several limitations. Firstly, the study had a small sample size which limits the transferability of our results to other hospital settings. As we did not interview a clinician from all departments with HRC, we cannot rule out the possibility of missing additional themes or barriers. Emails inviting staff members to participate were sent out via Department Heads and so we were unable to document the number of staff members that declined and the reason. Furthermore, participants in our study had generally positive attitudes towards the influenza vaccine. HCWs who are highly disengaged with the value of influenza immunisation are unlikely to volunteer to be involved. In a recent survey of Sydney pediatricians, less than half (48%) considered the influenza vaccine useful [15]. Thus, ‘negative cases’ warrant further investigation to better characterise the perspectives of HCWs who may be reluctant to promote or provide the vaccine. Lastly, we did not undertake interviews with parents and so the comments around parental acceptance/concerns about the influenza vaccination are based on the perceptions of the HCWs. Despite this, we feel that the findings of this study are relevant to other hospital sites that don’t currently have dedicated immunisation clinics but who could consider enhancing their role in vaccine promotion/delivery.

## Conclusion

HCWs play a key role in influenza vaccine uptake in HRC. Active promotion by HCWs and on-site delivery of the vaccine may facilitate immunisation in the hospital setting. Conversely, HCWs may refrain from vaccine conversations due to a lack of knowledge and the presence of competing priorities. Staff members must be considered when designing hospital-based interventions to ensure they are appropriate for the local health setting. A ‘bundle approach’ that targets HCWs and parents is likely required to achieve optimal vaccine coverage at this hospital. This would involve: 1) educating HCWs on the current recommendations for HRC; 2) motivating HCWs to discuss the influenza vaccine in all consultations; 3) increased engagement with parents, and 4) additional resources to allow more streamlined vaccine delivery (e.g. additional nurses).

## Supplementary information

**Additional file 1.** Interview guide.

## Data Availability

De-identified data is available upon request from the corresponding author.

## Abbreviations

GP: General practitioner
HCW: Healthcare worker
HRC: High-risk children
