# Supplementary material for: Exploring strategies to promote influenza vaccination of children with medical comorbidities: the perceptions and practices of hospital healthcare workers
Source: BMC Health Serv Res. 2019 Nov 29;19:911. doi: 10.1186/s12913-019-4742-5 (PMC6883556; doi:10.1186/s12913-019-4742-5)
Supplement: Supplementary file 1 — Additional file 1. Interview guide. [file 12913_2019_4742_MOESM1_ESM.docx]

Does the issue of immunisation ever come up during your consultations with medically at-risk patients? Who usually initiates the conversation?

How much do you know about the current influenza vaccine recommendations?

What do you think about the recommendations?

Do you normally recommend the influenza vaccine to your patients? If so, what reactions do you receive from parents? Do you feel comfortable in addressing questions about immunisation?

In Australia, most children get vaccinated in primary care. What are your thoughts on kids getting vaccinated at the hospital?

Do you envisage there being any barriers to prompting or delivering more immunisation services in the hospital setting?

What strategies would support you in increasing your level of engagement with promoting influenza vaccine for at-risk kids

Can you describe the processes used here at the hospital to promote and deliver immunisation services to patients?
